# Development and preliminary results on the feasibility of a renal diet specific question prompt sheet for use in nephrology clinics

**DOI:** 10.1186/s12882-019-1231-3

**Published:** 2019-02-12

**Authors:** Kelly Lambert, Tsz Kwan Lau, Sarah Davison, Holly Mitchell, Alex Harman, Mandy Carrie

**Affiliations:** 1 Department of Clinical Nutrition, Wollongong Hospital, Illawarra Shoalhaven Local Health District, Level 5 Block C, Crown Street, Wollongong, NSW 2500 Australia; 2 School of Medicine, Faculty of Science, Medicine and Health, University of Wollongong, Northfields Ave, Wollongong, NSW 2522 Australia

**Keywords:** Patient-centered care, Self-management, Chronic kidney disease, Health communication, Feasibility study, Dietitian, Nephrology

## Abstract

**Background:**

Adherence to the diet prescription for chronic kidney disease is suboptimal. Interventions to improve dietary adherence suggest that improving communication between the patient and the health professional is fundamental to improving outcomes. Providing patients with a question prompt sheet prior to the consultation has been demonstrated to be an effective method for improving communication between patient and health professional. In the absence of a renal diet specific version, the aims of this study were to develop and test the feasibility of a renal diet specific question prompt sheet for use in nephrology clinics.

**Methods:**

Phase one utilized social listening methodology, online content analysis and clinic observations to obtain an extensive list of frequently asked questions about the renal diet. Following refinement with health professionals, the draft question prompt sheet was then sent in Phase two to patients one week prior to their scheduled consultation with the renal dietitian. Feedback was obtained from patients, carers and dietitians using semi structured interviews post clinic consultation. Quantitative data was analyzed using counts and proportions, while free text responses were analyzed thematically.

**Results:**

A total of 769 unique renal diet related questions were reduced to an 18-item question prompt sheet. Feedback from thirteen patients (six males), six carers and six dietitians involved in the preliminary feasibility study was overwhelmingly positive. The majority of patients found the question prompt sheet to be easy to understand and agreed it facilitated communication with the dietitian. All participants agreed that they would recommend use of question prompt sheet to other patients. Suggestions for future use included health professional training in use of the sheet, particularly about how to help patients prioritize their most important questions.

**Conclusions:**

The 18-item renal diet question prompt sheet developed in this preliminary study appears to be a feasible tool for use in nephrology consultations especially by dietitians. Further research quantifying the impact on question asking and patient centeredness should be undertaken. In addition, user testing with patients from culturally diverse and low literacy backgrounds would be useful.

**Electronic supplementary material:**

The online version of this article (10.1186/s12882-019-1231-3) contains supplementary material, which is available to authorized users.

## Background

Chronic Kidney Disease (CKD) is a common chronic illness with an enormous global public health burden [1]. Dietary modification is a critical part of the management of CKD [2] because it can slow progression of CKD to End Stage Kidney Disease (ESKD) [3]; ameliorate the complications of CKD and ESKD [4–7], and increase survival [8, 9]. One of the major challenges faced by patients with CKD when learning about the renal diet is to navigate the changes in the dietary prescription as CKD progresses, and to balance these dietary changes with the needs of other comorbidities such as diabetes. This complexity has often been cited as one the main reasons for the feelings of frustration, bewilderment and confusion about the diet expressed by patients with CKD and their carers [10, 11]. Given the challenges imposed by the renal diet, it is unsurprising that dietary adherence remains around 32% [12] .

Interventions to improve dietary adherence, particularly those in patients with CKD suggest that improving communication between the patient and the health professional is fundamental to improving outcomes [13]. Studies on dietary adherence in ESKD repeatedly report that patients and carers do not know what questions to ask health professionals, and that ineffective communication is a barrier to adherence [11]. However, strategies to improve communication, particularly those conversations regarding dietary modification are not well described. Interventions from other health care contexts suggest that use of a question prompt sheet (QPS, also known as question prompt list) is one potential tool that could be utilised. The QPS is a list of questions provided to the patient before the consultation [14]. The QPS can be used to help patients and carers initiate conversations in the consultation that are focused on their information needs, as well prepare them for what may be discussed. In addition to being well accepted by patients [15], previous research suggests that a QPS is also effective at increasing question asking [14], recall of information [16], patient knowledge [17], and patient anxiety [18].

A CKD specific question prompt sheet has recently been developed [19]. It consists of 31 questions about treatment of CKD, including three general questions about diet. While patients were extensively involved in the development of the QPS, it lacks sufficient detail about diet for use by health professionals to discuss renal diet related concerns. Given that patients and carers want explicit information and signposting about the renal diet [20], and there is no alternative QPS developed to facilitate diet related discussions, the aims of this study were to develop a renal diet specific QPS for use in nephrology clinics to facilitate diet related communication between patients and the health professional.

## Methods

**Fig. 1:**
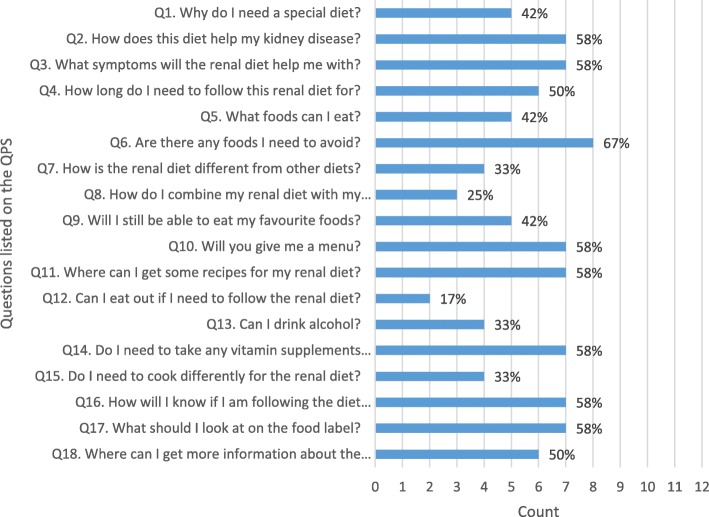
Frequency of questions selected on the renal diet question prompt sheet

This study is comprised of two phases. Phase one utilised content analysis of online information and forums, observations of questions asked by patients in renal diet clinics and expertise from health professionals to construct the final QPS (Fig. 1). All questions from clinic observations and online sources were recorded verbatim to gain an insight into the terminology commonly used by patients and carers. In Phase two, the feasibility of the QPS from the perspective of health professionals and patients was also evaluated. Ethical approval for the study was provided by the joint University of Wollongong Illawarra Shoalhaven Local Health District Human Research Ethics Committee (Approval Number 2018/050).

### Phase 1: Creation of the QPS

#### Renal diet questions raised by patients on social media

This phase utilised the method of social listening to obtain information about commonly asked renal diet questions. Social listening is a new research method, and involves tracking conversations on social media around a particular topic using keywords or phrases [21]. Previous research by our group suggest that social media and online forums are frequented by our patient population with CKD [20]. We therefore hypothesised that this method could provide useful insights into questions that patients or carers may have about the renal diet in the absence of immediate access to a health professional.

To conduct this analysis, a manual count of renal diet related questions on the two large CKD related Facebook groups (‘Chronic Kidney Disease Stage 3’ and ‘Kidney-Friendly Cooking’) were undertaken from 22 January to 23 February 2018. These groups contain in excess of 14,700 members. Facebook is the primary social media platform of choice for most adults, with more than two thirds of Americans using Facebook, and more than three quarters accessing it on a daily basis [22]. All posts on the two groups were viewed in reverse chronological order from the date of group creation to present, and all renal diet related questions were recorded verbatim.

#### Content analysis of renal diet questions asked online

To extract a list of frequently asked questions on the search engine Google, the ‘People also ask’ function was utilised. This expandable drop down function appears following the entry of a search term into the search engine, and contains questions in response to the most commonly searched terms [23]. The six keywords entered into Google are shown in Table 1 and include: “chronic kidney disease diet” or “CKD diet” or “renal diet”. These terms are based on previous research analyzing online renal diet information [24]. Google was chosen because it is the most frequently used search engine globally, and holds 92.3% of the global search engine market share [25]. The questions obtained from Google were categorised according to a predefined coding system. This system was developed by the research team based on clinical experience and reflections by the research team of frequently asked renal diet related questions (see Additional file 1).

**Table 1:** Keywords used on Google search engine to explore commonly asked questions

Keywords	Example questions identified
“chronic kidney disease diet” or “CKD diet”	Which foods are good for chronic kidney disease? What foods to avoid if you have kidney problems? How do I lose weight with chronic kidney disease? Can you eat peanut butter if you have kidney disease / on a renal diet?
“renal diet”	What do you eat on a renal diet? What not to eat on a renal diet? Why would a patient be on a renal diet? Can you eat chocolate on a renal diet?
“what foods to eat when you have chronic kidney disease”	Is eggs good for kidney patients? Which juice is best for kidneys? What vitamins are good for the kidneys?
“what foods to avoid when you have chronic kidney disease”	Can you eat potatoes with kidney disease? What food is bad for high potassium? Is drinking water good for the kidneys?
“can ckd patients …”	… eat tomatoes … drink coffee
“can renal patients …”	… eat sweet potatoes … have milk

In addition to the Google function, the questions placed on eight popular online renal diet forums were also interrogated. This type of research method provides rich qualitative data. It also is appropriate for use in this study because patients and carers with kidney disease are known to frequent online forums, particularly if access to a renal dietitian is difficult or they are newly diagnosed and have not yet accessed health professional services [20]. The forums and Facebook groups accessed and examples of the types of questions posted are contained in Additional file 2.

#### Observations of renal diet clinics

Additional information to inform the development of the renal diet QPS was obtained from observations made at four renal dietitian clinics in two hospitals. The type and total number of renal diet related questions raised by patients and carers were recorded verbatim. Written consent was obtained from patients to observe the clinics and record observations.

#### Refinement of the QPS

A total of 769 unique renal diet related questions were identified from all sources. The questions obtained were categorised according to a predefined coding system developed for the purposes of this research (Additional file 1). Repetitive questions and recurring patterns in the questions were identified and reduced to common themes. “For example, questions about specific food items were reduced to a common question titled” “what can I eat?”. This resulted in an 18 item draft QPS divided into four categories: common questions about the renal diet; about the diet; monitoring my health on the diet; and finding more information about the renal diet.

The next step in the refinement of the draft QPS was review by health professionals. The review panel consisted of two renal dietitians and three experienced non renal dietitians. In addition, three experienced renal dietitians were recruited from professional networks to provide additional feedback. In addition to reviewing the content, question wording, and layout, specific feedback was also elicited regarding how the QPS might be best implemented for its intended use. One additional component about how to use the QPS was added based on this feedback: “Please bring this sheet with you to the consultation with the Dietitian. They will discuss the answers to these questions, as well as any other questions you may have”.

A readability assessment was conducted using the online readability calculator recommended by our health district when developing patient information (http://www.readabilityformulas.com/free-readability-formula-tests.php). In brief, the calculator uses seven different readability calculators to estimate the level of education required to comprehend the written information.

The final QPS consisted of 18 items and includes an explanation of how to use the QPS (Table 2). The readability of the final version was estimated at 4th to 5th grade level and is considered easy to read.

**Table 2:** Final Renal Diet Question Prompt Sheet

Dear Patient, This information sheet has been sent to you by the Renal Dietitian. Please read through the questions before your consultation and make a tick ✓next to questions that are important to you. Think about other questions about the diet that you might have and write them on the other side of this sheet. Please bring this sheet with you to the consultation with the Dietitian. They will discuss the answers to these questions, as well as any other questions you may have.	
Common questions about the renal diet □ Why do I need a special diet? □ How does this diet help my kidney disease? □ What symptoms will the renal diet help me with? □ How long do I need to follow this renal diet for?	
About the diet □ What foods can I eat? □ Are there any foods I need to avoid? □ How is the renal diet different from other diets? □ How do I combine my renal diet with my diabetic diet? □ Will I still be able to eat my favourite foods? □ Will you give me a menu? □ Where can I get some recipes for my renal diet? □ Can I eat out if I need to follow the renal diet? □ Can I drink alcohol? □ Do I need to take any vitamin supplements when I am on the renal diet? □ Do I need to cook differently for the renal diet?	
Monitoring my health on the renal diet □ How will I know if I am following the diet correctly?	
Finding more information about the renal diet □ What should I look at on the food label? □ Where can I get more information about the diet?	
My own questions for the Dietitian:	

### Phase 2: Feasibility of the QPS

For this phase, a convenience sample of all consecutive patients attending the three public hospital based renal dietitian clinics in one health district over a 4 week period were eligible for inclusion in the feasibility study. There were four exclusion criteria for the study: (i) patients unable to give informed consent (ii) patients referred to the clinic with stage 5CKD for conservative management (iii) patients and/ or carers who did not wish for their consultation to be audio recorded (iv) and dietitians who did wish for their consultation to be recorded. No dietitians were excluded, and one carer declined for the consultation be audio recorded and was excluded from the study.

The final QPS was addressed to the patient and sent by mail at least 1 week prior to the consultation. Instructions to complete the QPS and to bring it to the clinic consultation were included on the final QPS (Table 2). Although dietitians were aware patients had been provided with a QPS, no specific instructions were provided to the dietitians in the clinics about how to use the QPS. This was intentional and used to ascertain if patients and carers initiated questions with the dietitian using the QPS, and to obtain feedback about potential barriers to use and suggestions about how best to utilise the QPS in a pragmatic setting. Feedback about the QPS was obtained from patients and carers immediately following the consultation by a member of the research team (KL or TKL). The feedback obtained from patients consisted of eight questions adapted from work by Arthur et al. [15] in their project utilising the QPS in an outpatient palliative care setting. The evaluation (Additional file 3) was intended to obtain information about the content as well as use of the QPS within the clinic. Response ratings were on a five-point Likert scale (strongly disagree to strongly agree). Space was also provided to include free text comments about the QPS. Feedback from the clinic dietitians (*n* = 6) was obtained by undertaking individual semi structured interviews using six open ended questions (Additional file 4).

During the four-week study period, thirteen patients and six carers attended the clinics (attendance rate of 87%). The median age of patients was 60 years (Interquartile range: 54–70). Six patients were male. Nine of the 13 participants had CKD stage 3–5; one was on dialysis, one had received a transplant and 2 were early CKD (stage 1 or 2). Of the 13 patients, only two had received dietary advice previously (one for hemodialysis diet advice but was referred for post transplantation advice; one for low potassium diet advice but was referred in the study period for pre transplantation weight loss advice). The main reasons for referral were weight management (*n* = 7) and nutrition support (*n* = 3). Of the thirteen participants, 12 brought the QPS with them to clinic as directed. The most frequently selected item on the QPS by patients were: “Are there any foods I need to avoid?” (67% of participants, Fig. 1). The least frequently selected item on the QPS was “Can I eat out if I need to follow the renal diet?” (17%). An additional 8 questions were recorded on the QPS by patients. Three of these questions related to where and how to obtain more information (especially online) if required. Two questions were non diet related (sleep and phantom pain, safety of exercise).

Most participants either agreed or strongly agreed that the QPS helped them communicate with the dietitian (Tables 3, 92%); contained easy to understand information (100%); would recommend using it to other patients (100%); and agreed that it helped them to think of questions that they had not thought of before (100%). Of note, two patients reported that they would not use the QPS to help them write down questions in the future before they saw the health professional. When questioned further, these patients iterated that this was because they believed all questions were answered adequately by the dietitians during the consultation and they had no further questions.

**Table 3:** Patient Evaluation of the QPS in renal dietetic outpatient clinics

Question	Strongly disagree (%)	Disagree (%)	Undecided (%)	Agree (%)	Strongly agree (%)
Do you think the QPS helped you communicate with the Dietitian?	0	0	1 /12 (8%)	6 /12 (50%)	5 /12 (42%)
Do you think the information in the QPS was easy to understand?	0	0	0	6 /12 (50%)	6 /12 (50%)
Do you think the information in the QPS was about right?	0	0	0	8 /12 (67%)	4 /12 (33%)
Do you think you would recommend the QPS to others?	0	0	0	5 /12 (42%)	7 /12 (58%)
Would you use the QPS or something similar to write down questions in the future before you see the Dr. or Dietitian?	0	2 /12 (17%)	0	5 /12 (42%)	5 /12 (42%)
Do you think the QPS helped you to think of questions you had not thought of before?	0	0	0	7 /12 (58%)	5 /12 (42%)
Overall how satisfied are you with the Dietitian visit today?	0	0	0	2 /12 (17%)	10 /12 (83%)
Do you have any other comments about the QPS?					

Additional qualitative feedback from patients was also overwhelmingly positive. For example, one patient commented that it was very valuable because “a lot of people forget what they want to ask and it can be used to their jog memory” (Patient 12). Similarly, several commented that they appreciated the design of QPS where patients could simply tick boxes rather than writing down questions themselves as one patient articulated “you don’t know what to ask” (Patient 6). The content of the QPS was found to be valuable for a number of patients and carers who were unsure why they had been referred to the dietitian. The questions in the QPS were reported to be useful for clarifying how the dietitian could be helpful to them: “it gave you something to reference … you don’t know what to ask or think about and it helped me learn what the special kidney diet was about and (to) see if you are on the right track” (carer of Patient 7). Changes to the QPS suggested by patients included changing the wording from: “renal diet” to “special diet”; including a date; and ensuring it was sent at least 1 week prior. The single patient who did not bring the QPS reported that it was because it had only arrived in the mail that day.

Feedback from dietitians about the QPS was also positive, and several important recommendations about the use of the QPS were provided. All dietitians agreed that the QPS was a valuable tool and that it facilitated question asking in the clinic: “it made patients who were previously passive more proactive in question-asking” (Dietitian 4). However, several expressed confusion about how to use it with patients: “the patient held onto it and I think they were half expecting me (the dietitian) to launch straight into it” (Dietitian 1) and “the patient filled it in, brought it along and put it onto the table, and I was a bit perplexed about what to do with it” (Dietitian 6). All dietitians agreed that it did not extend the consultation time. However, the dietitians also described feeling confused about how to address patient concerns when a large number of questions on the QPS were selected (as had been observed in some cases). While the question: “Will I get a menu?” was a popular question for patients, dietitians expressed reluctance about providing this resource to patients. The final recommendation from dietitians was for training about how to use the QPS, and about how to accurately answer specific questions (foods to avoid, vitamin supplements, and where can I get more information about the diet). Two dietitians also suggested it might be useful to prompt patients to read through the QPS in the waiting room prior to their consultation with the dietitian.

## Discussion

Adherence to treatment is one of the most effective method for improving health outcomes [26]. Alternative approaches to patient education that enhance communication may help dietitians to empower patients to better understand and adhere to the renal diet [27]. The intent of this study was therefore to develop and trial the feasibility of a renal diet specific QPS for use in nephrology clinics. The study has yielded three key findings that can be used by clinicians and researchers to facilitate question asking and encourage greater engagement with health professionals regarding renal diet discussions.

Firstly, feedback about the content of the renal diet QPS suggests that when used in the clinic setting it facilitates patients and carers to meet their unmet renal diet information needs. All 18 questions on the QPS were selected by patients and their inclusion was based on an extensive review of the questions posed by people with kidney disease on online forums and raised in the clinic. The nature of the questions and the number of items included are similar to QPS research in other settings [14, 16]. However, a QPS is unable and should not replace adequate communication with a health professional to describe the nuances of the renal diet. The QPS should be used as an adjunct to good communication, and to help tailor the session with the patient to address their concerns. Whilst, information that is important to patients is more likely to be remembered [28], additional strategies are required to check patient recall and understanding [29]. This could include use of ‘teachback’, an evidence based communication strategy used to evaluate patient comprehension of important concepts [30].

The high level of satisfaction expressed by patients, carers and dietitians with the QPS suggests that it is a feasible and a valuable tool for use in nephrology clinics. Previous research has suggested that providing the QPS immediately prior to the clinic is preferred [14]. Previous studies also report low usage rates when a QPS is sent a week or more prior to the consultation [14]. This contrasts with the current study, where patients preferred to receive it in this manner, and spend time at home reading and thinking about the questions and preparing for the session in advance. Further studies suggest that endorsement of the QPS by the health professional [28], and adequate explanations on how to use the QPS also increased usage [31, 32]. Feedback from patients in this study also indicated that the QPS may be less valuable for patients at review consultations, although all review patients in this study indicated they wanted to keep the QPS as a memory aid about important concepts. We believe that provision of the QPS at important points in the CKD journey [33], such as when first diagnosed or at change of renal replacement therapy may be optimal. Further research exploring return rates and whether endorsement from the health professional impacts use of the QPS in the dietetic setting would be useful.

Few studies have described the specific need for health professional training when using a QPS. Anxiety about the use of the QPS was an important element raised by dietitians in this study. Several studies using the QPS have suggested that additional communication training of health professionals may be beneficial [34, 35]. This may include wording about how to acknowledge that a patient has brought the QPS to clinic. Dimoska et al. [36] have also provided useful wording about how health professionals can phrase their endorsement for use of the QPS. However little guidance is provided about how health professionals should structure the consult to best meet the needs of the patients. It is suggested dietitians proactively discuss with patients and carers the most important questions on the renal diet QPS that they would like addressed in order to reduce cognitive demand [37], prevent information overload and aid knowledge retention [38]. It was apparent in this study, that having ready access to resources or appropriate ‘answers’ to some of the questions within the QPS would be beneficial for dietitians. This could include updated information about appropriate online renal diet information and sample meal plans. Other factors that have been identified that facilitate successful use of the QPS include local champions, dissemination via administrative staff in a timely manner, patient led use in the consultation [35], promotion about QPS use and availability of the QPS in multiple languages [33].

There are several strengths of this study. This includes the use of comprehensive searches to obtain common questions raised by patients and carers about the renal diet, and attention to health literacy principles when developing the wording within the tool. Feedback from patients was incorporated when refining the tool. The input from health professionals in this study is also an important strength, as there is a paucity of literature examining the impact of a QPS on the health professional. However, the limitations must also be acknowledged, and these include the small sample size of participants and short time frame of the study. Despite gaining useful qualitative feedback about the QPS, we failed to specifically ask who complete the QPS: the patient, carer or both. This requires further exploration. Research exploring QPS use in culturally diverse populations using interpreters and in those with low literacy would be useful. Future studies should also examine how use of the QPS may change over the CKD journey and with repeated use, and details about what patients perceive to be an appropriate timing and mode of delivery. Exploration of the use of the QPS with health professionals with varying levels of renal dietetic expertise and familiarity with QPS would also be informative. Furthermore, consideration should be given to the development of an ‘answer’ sheet to the renal diet QPS as this may potentially be useful for patients, their family and carers or non-renal health professionals. Interventions using the renal diet QPS should be conducted to quantify the impact on question asking and the patient centeredness of consultations. If effective, then adaptation of the renal diet QPS for use in other dietetic patient cohorts such as gastrointestinal, allergy or diabetes management contexts may be possible.

## Conclusions

Effective communication between the patient and health professional is central to improving health outcomes. The 18-item renal diet question prompt sheet developed in this study appears to be a feasible tool for use in nephrology consultations especially by dietitians. Further research quantifying the impact on question asking and patient centeredness should be undertaken. In addition, user testing with patients from culturally diverse and low literacy backgrounds would be useful.

## Additional files

Additional file 1: Coding category system for renal diet questions. (DOCX 15 kb)

Additional file 2: Forums and Facebook groups accessed and examples of questions. (DOCX 18 kb)

Additional file 3: Patient and carer evaluation form regarding the renal diet question prompt sheet. (DOCX 15 kb)

Additional file 4: Dietitian evaluation form regarding the renal diet question prompt sheet. (DOCX 14 kb)

## Abbreviations

CKD: Chronic Kidney Disease
ESKD: End Stage Kidney Disease
QPS: Question Prompt Sheet
