# Supplementary material for: Development and preliminary results on the feasibility of a renal diet specific question prompt sheet for use in nephrology clinics
Source: BMC Nephrol. 2019 Feb 12;20:48. doi: 10.1186/s12882-019-1231-3 (PMC6373020; doi:10.1186/s12882-019-1231-3)
Supplement: Supplementary file 1 — Coding category system for renal diet questions. (DOCX 15 kb) [file 12882_2019_1231_MOESM1_ESM.docx]

Supplementary material 1: Coding category system for renal diet questions

| Topics | Examples of questions / topic areas |
| --- | --- |
| Dietitian’s role in the patient journey | - What does a dietitian do or how can a dietitian help ? - When to see a dietitian and how frequently |
| Understanding CKD and medications | - Why or how to take medications (such as Resonium and phosphate binders) - Information or advice from other health professionals |
| Biochemistry | - What, why and how to manage (such as potassium and phosphate) - Potential consequences of not adhering to recommendations |
| CKD stages | - Changes in dietary guidelines at different CKD stages, on haemodialysis or peritoneal dialysis, and post-transplant - Dietary advice contradicting “healthy diet” or “diabetes diet” - Dietary advice on macronutrients, micronutrients and fluid intake, as well as why are they necessary or changing |
| What to eat, limit or avoid | - Food groups and examples - Discretionary food choices - Special diets (such as vegetarian and Mediterranean) |
| Fluid control | - What counts as fluid - What and how much to drink, limit or avoid - Thirst management - Specific beverages (such as alcohol, coffee and tea) |
| Nutrient and recipe analysis | - Nutrient content, food additives, label reading - Food exchanges - Portion control, frequency of consumption |
| Meal or snack ideas, recipes, free foods | - Cooking or food preparation methods, food swaps - Seasoning, sugar and salt substitute - Frozen meals, meal delivery or Meals on Wheels - What to eat on dialysis - Food choices while eating out or travelling - Party or holiday food choices |
| Safety and efficacy of supplements | - Oral nutrition support - Protein powder, vitamin and/or mineral supplements - Herbs |
| Other medical conditions | - Managing comorbidities (such as diabetes and hypertension) - Managing other medical conditions (such as celiac disease) - Priorities of dietary management - Managing conflicting dietary advice (if any) |
| Weight management | - Weight gain or loss |
| Symptom or condition management | - Managing appetite, hunger or cravings, compulsive thoughts about food or fluid - Managing symptoms (such as constipation, fatigue and itching) |
| Additional information | - Where to find evidence-based information - Useful mobile applications (such as tracking nutrient intake) |
