# Supplementary material for: Development and preliminary results on the feasibility of a renal diet specific question prompt sheet for use in nephrology clinics
Source: BMC Nephrol. 2019 Feb 12;20:48. doi: 10.1186/s12882-019-1231-3 (PMC6373020; doi:10.1186/s12882-019-1231-3)
Supplement: Supplementary file 2 — Forums and Facebook groups accessed and examples of questions. (DOCX 18 kb) [file 12882_2019_1231_MOESM2_ESM.docx]

Supplementary material 2: Forums and Facebook groups accessed and examples of questions

| DaVita - kidney diet tips  <http://forums.davita.com/forum/diet-nutrition/the-kidney-diet/kidney-diet-tips-q-a-s> | - How is it possible to get at least 1200 mg of calcium a day while limiting protein, potassium and sodium? - In similar foods (tortillas for instance) which is the best choice - lower phosphorous with higher potassium or higher phosphorous with lower potassium? - Is Gatorade a beverage that might affect the kidneys and in what way if so |
| --- | --- |
| DaVita - dialysis diet  <http://forums.davita.com/forum/diet-nutrition/the-kidney-diet/dialysis-diet> | - For a patient in dialysis: White rice or Brown? Regular noodles or wheat? White potatoes or red? - Where can I get salt free seasoning? Salt substitute ? - Can you help me give some tips for a proper guide on preparing food for my mom ? |
| DaVita - CKD diet  <http://forums.davita.com/forum/diet-nutrition/the-kidney-diet/the-ckd-diet> | - What are good substitutes for salt for people with CKD? - Is there a list of the bad things not to eat? - I found a recipe for cashew nut cream cheese. Is this a safe alternative to dairy products? |
| DaVita - dining out  <http://forums.davita.com/forum/diet-nutrition/the-kidney-diet/dining-out> | - Can anyone give me any kidney-friendly fast-food ideas that are also low in sodium? - How to get enough calories in the day without going over phosphorus limit? - Does anyone know what foods are best with someone with chronic kidney disease and what restaurants are best to go too? |
| DaVita - DaVita diet helper  <http://forums.davita.com/forum/diet-nutrition/the-kidney-diet/davita-diet-helper> | - My husband's recommended daily intake of meat protein is 6 oz. How do you measure it? Did you buy a scale? Any good web resources? - Can you please give me help with some plain suggested meals, recipes ? - Would appreciate Vegan CKD Recipe websites, or cookbook suggestions. |
| Your Kidney Forum (Kidney Health Australia)  <http://forum.kidney.org.au/> | - Is there anything we can try to do to slow down the swelling and manage his fluid intake |
| Medical conditions and fasting  <https://thefastdiet.co.uk/forums/forum/body/medical-conditions/> | - Is there any reason I should not do the 5:2 diet? - Hi, I would like to know if anyone else has had success with intermittent fasting and IgA nephropathy or CKD ? |
| Active Low-Carber Forums  <http://forum.lowcarber.org/index.php>? | - I have Stage 3 CKD, I have read that a low carb diet is not advised ? - Is there any truth to the fact of prolonged stay on Atkins can cause future kidney problems ? - Has anyone ever started out on the LCHF diet and had either stage 1 or stage 2 kidney disease at the same time? Did you have to keep the protein low? |
| Diabetes.co.uk  <https://www.diabetes.co.uk/forum/threads/stage-4-ckd-and-gfr-levels.51358/> | - I am type 1 diabetic with stage 4 CKD. How do I lose weight ? - Is there anything I can do with my diet to get my numbers up? |
| HealingWell.com  <https://www.healingwell.com/community/default.aspx?f=27> | - I have been on a Paleo diet for four months and I think my animal protein intake has really increased my Creatinine levels – is this true ? - I am wondering if vegetable protein is much better than animal protein for kidney sufferers? - The doctor has required my wife to eat foods with little (or preferably) to zero potassium. The Bigger Problem: He's given her many lists of what NOT to eat, but can't seem to give her a list of what she CAN eat. |
| Kidney Patient Guide  <http://www.kidneypatientguide.org.uk/forum/viewforum.php?f=2> | - Could my rapid weight loss have affected my GFR, creatinine, & BUN numbers? - I'm reading stage 3 ckd patients should adopt a low-protein, low phosphorus diet. If anyone could contribute any reliable diet tips on how to accomplish this, I would be most grateful. - I had a transplant and I am doing very well post-transplant. The only downside with me is the weight gain. I want to start taking some supplements and introducing a whey protein into my diet to encourage me to build muscle and lose fat – is this safe ? |
| Patient.info  <https://patient.info/forums/discuss/browse/kidney-failure-and-ckd-1300> | - Is moderate consumption of wine is good for people with CKD? - I wondered if any of you would be so kind as to share some of your favorite renal friendly recipes - I have been on diuretics … I also have to take warfarin. By the time I rule out all of the warfarin no-no's of my diet and then look at a low-protein diet, I keep thinking my goodness what's left.  Do you all know of any books that would give me a good start? |
| Fresenius  <http://www.topix.com/forum/com/fms> | - What is there to eat or drink while on the machine? |
| Facebook Chronic Kidney Disease stage 3  (public group: 14,327 members) | - Can anyone recommend a breakfast cereal with low salt or phosphorous? - What foods to eat & avoid eating when you are ckd stage 4 patient? - What foods to eat & avoid eating before, during, & after hemodialysis treatment? |
| Kidney-Friendly Cooking  (public group: 460 members) | - My husband is diabetic and has Stage 4 kidney disease. What can I give him to eat that is kidney friendly? - Husband on PD and we just can't figure out lunch! Help!!! - Anyone have a good biscuit recipe ? |
