# Supplementary material for: Development and preliminary results on the feasibility of a renal diet specific question prompt sheet for use in nephrology clinics
Source: BMC Nephrol. 2019 Feb 12;20:48. doi: 10.1186/s12882-019-1231-3 (PMC6373020; doi:10.1186/s12882-019-1231-3)
Supplement: Supplementary file 3 — Patient and carer evaluation form regarding the renal diet question prompt sheet. (DOCX 15 kb) [file 12882_2019_1231_MOESM3_ESM.docx]

Supplementary material 3: Patient and carer evaluation form regarding the renal diet question prompt sheet

1. Did you bring the question prompt sheet to the visit? If no – why not ?
2. Do you think the QPS helped you communicate with the dietitian ?

| Strongly disagree | Disagree | Undecided | Agree | Strongly agree |
| --- | --- | --- | --- | --- |
| Other comments | | | | |

1. Do you think the information in the QPS was easy to understand ?

| Strongly disagree | Disagree | Undecided | Agree | Strongly agree |
| --- | --- | --- | --- | --- |
| Other comments | | | | |

1. Do you think the information in the QPs was about right ?

| Strongly disagree | Disagree | Undecided | Agree | Strongly agree |
| --- | --- | --- | --- | --- |
| Other comments | | | | |

1. Do you think you would recommend the QPS to others ?

| Strongly disagree | Disagree | Undecided | Agree | Strongly agree |
| --- | --- | --- | --- | --- |
| Other comments | | | | |

1. Would you use the QPS or something similar to write down questions in the future before you see the Dr or dietitian ?

| Strongly disagree | Disagree | Undecided | Agree | Strongly agree |
| --- | --- | --- | --- | --- |
| Other comments | | | | |

1. Do you think the QPS helped you to think of questions you had not thought of before ?

| Strongly disagree | Disagree | Undecided | Agree | Strongly agree |
| --- | --- | --- | --- | --- |
| Other comments | | | | |

1. Overall how satisfied are you with the dietitian visit today ?

| Strongly disagree | Disagree | Undecided | Agree | Strongly agree |
| --- | --- | --- | --- | --- |
| Other comments | | | | |

| Do you have any other comments about the QPS ?  🞏 send it to me earlier ?  🞏 give it to me in the waiting room  🞏 add more questions such as …  🞏 other comments |
| --- |
