# Supplementary material for: Development and preliminary results on the feasibility of a renal diet specific question prompt sheet for use in nephrology clinics
Source: BMC Nephrol. 2019 Feb 12;20:48. doi: 10.1186/s12882-019-1231-3 (PMC6373020; doi:10.1186/s12882-019-1231-3)
Supplement: Supplementary file 4 — Dietitian evaluation form regarding the renal diet question prompt sheet. (DOCX 14 kb) [file 12882_2019_1231_MOESM4_ESM.docx]

Supplementary material 4: Dietitian evaluation form regarding the renal diet question prompt sheet

| Can you describe how you observed the QPS was used during the consultation ?   - - Did they bring it ?   - What did they do with it ?   Who referred to it ? patients or carers ? |
| --- |
| Do you think the QPS helped patients communicate their questions or concerns with you ? |
| Did the QPS extend consult time ? |
| Did the QPS affect other aspects of the consultation ?   - - Flow of info gathering   - Process of info giving   - Anything else |
| Did you have any feedback on QPS |
| Do you think the QPS would be valuable ongoing addition to the process of providing care to renal patients in the outpatient setting |
